# In vitro drug testing using patient-derived ovarian cancer organoids

**DOI:** 10.1186/s13048-024-01520-2

**Published:** 2024-10-02

**Authors:** Lin-Yu Chen, Yu-Ting Chou, Phui-Ly Liew, Ling-Hui Chu, Kuo-Chang Wen, Shiou-Fu Lin, Yu-Chun Weng, Hui-Chen Wang, Po-Hsuan Su, Hung-Cheng Lai

**Affiliations:** 1 Department of Obstetrics and Gynecology, Shuang Ho Hospital, Taipei Medical University, New Taipei City, 23561 Taiwan; 2 Department of Pathology, Shuang Ho Hospital, Taipei Medical University, New Taipei City, 23561 Taiwan; 3 Department of Pathology, School of Medicine, College of Medicine, Taipei Medical University, Taipei, 11031 Taiwan; 4 Department of Obstetrics and Gynecology, School of Medicine, College of Medicine, Taipei Medical University, Taipei, 11031 Taiwan; 5 Translational Epigenetics Center, Shuang Ho Hospital, Taipei Medical University, New Taipei City, 23561 Taiwan; 6 Department of Obstetrics and Gynecology, Tri-Service General Hospital, National Defense Medical Center, Taipei, 11490 Taiwan; 7 College of Health Technology, National Taipei University of Nursing and Health Sciences, Taipei, 11219 Taiwan

**Keywords:** Patient-derived organoids, Drug testing, Ovarian cancer, Precision medicine

## Abstract

**Background:**

Ovarian cancer is the most lethal gynecological cancer. As the primary treatment, chemotherapy has a response rate of only 60–70% in advanced stages, and even lower as a second-line treatment. Despite guideline recommendations, which drugs will be most effective remains unclear. Thus, a strategy to prioritize chemotherapy options is urgently needed. Cancer organoids have recently emerged as a method for in vitro drug testing. However, limited clinical correlations have been assessed with test results from cancer organoids, particularly in gynecological cancers. We therefore aimed to generate patient-derived organoids (PDOs) of ovarian cancer, to assess their drug sensitivities and correlations with patient clinical outcomes.

**Methods:**

PDOs were generated from fresh tumors obtained during surgical resection, which was then cultured under matrix gel and appropriate growth factors. Morphological and molecular characterization of PDOs were assessed by phase contrast microscopy and paraffin-embedded histopathology. Expressions of PAX8, TP53, WT1, CK7, and CK20 were tested by immunohistochemical staining and compared with parental tumor tissues and the human protein atlas database. PDOs were subjected to in vitro drug testing to determine drug sensitivity using Titer-Glo^®^ 3D Cell Viability Assay. PDO viability was measured, and area under the curve calculated, to compare responses to various compounds. Correlations were calculated between selected patients’ clinical outcomes and in vitro drug testing results.

**Results:**

We established 31 PDOs. Among them, 28 PDOs can be expanded, including 15, 11, and 2 from ovarian, endometrial, and cervical cancers, respectively. The PDOs preserved the histopathological profiles of their originating tumors. In vitro drug testing of 10 ovarian cancer PDOs revealed individual differential responses to recommended drugs, and interpersonal heterogeneity in drug sensitivity, even with the same histology type. Among four patients who were platinum sensitive, resistant, or refractory, PDO drug responses correlated well with their clinical courses.

**Conclusion:**

In vitro drug testing using ovarian cancer organoids is feasible and correlates well with patient clinical responses. These results may facilitate development of precision chemotherapy and personalized screening for repurposed or new drugs.

**Supplementary Information:**

The online version contains supplementary material available at 10.1186/s13048-024-01520-2.

## Introduction

Epithelial ovarian cancer has remained among the most lethal gynecological cancers for decades, in part because it lacks recognizable physical symptoms before the tumor becomes enlarged or disseminated. Ovarian cancer detection is thus more likely at advanced stages (III or IV) with metastasis. In clinical practice, doctors follow established guidelines to treat patients with ovarian cancer, using surgery combined with chemotherapy as the most common approach. First-line chemotherapy relies on taxanes and platinum-based agents. The overall response rate to frontline chemotherapy by patients with advanced-stage ovarian cancer is 70–80% [1]. In patients with recurrent ovarian cancer, the response rate is lower. For patients with platinum-sensitive ovarian cancer, response rates to platinum-based combination chemotherapy range from 27 to 65%, whereas in platinum-resistant ovarian cancer, response rates to second-line chemotherapy are 10–30% [1]. There is currently no clear consensus or established methods regarding second- and third-line agents. Many patients with ovarian cancer experience ineffective conventional chemotherapy, despite administration of guideline-recommended drugs. Precision medicine is thus gaining importance in cancer therapies, but its application in ovarian cancer treatment remains at an early development stage.

Identifying the specific genetic characteristics of a tumor prior to treatment can guide physicians in developing a personalized approach, potentially leading to improved outcomes and fewer side effects. However, it is important to note that while 20–25% of women with ovarian cancer have a known genetic predisposition to the disease (e.g., breast cancer susceptibility gene (BRCA gene) mutations, other hereditary cancer syndromes), these factors do not guarantee response to a targeted therapy [1, 2]. For example, in the SOLO-3 clinical trial, up to 30% of patients with BRCA1/2 mutations did not respond to olaparib, a poly ADP-ribose polymerase (PARP) inhibitor, and 4% experienced disease progression despite treatment [2]. In the KEYNOTE-158 clinical trial, only 5 of 15 patients with high microsatellite instability/mismatch repair-deficient ovarian cancer had a tumor response to pembrolizumab [3]. These results suggest that tumors are heterogeneous and that genetic markers alone are insufficient for precision treatment. As a next step, one option may be in vitro drug testing using individual tumor cells.

The idea of drug testing as a surrogate for personalized chemotherapy has held appeal for many years. Primary cell culture cannot recapitulate the complexity of the tumor microenvironment; thus, drug testing has shown low relevance to clinical therapeutic responses [4, 5]. The patient-derived xenograft (PDX) model is a tumor environment system in which mouse-tested drug efficacy is now considered more relevant to clinical responses. However, its efficiency is relatively low [5, 6]. Establishing a PDX model means low transplant success rate, time-consuming and expensive test cycles [4–6]. Furthermore, PDX models are typically established in immunodeficient mice, to prevent human tumor cell rejection, and thus fail to capture the crucial tumor–immune system interactions. All of these factors hinder this model’s clinical application.

A novel three-dimensional (3D) culture technology has been used to grow a structure consisting of various organ-specific cell types, called an ‘organoid’ [7]. Compared with traditional cell culture models, patient-derived organoids (PDOs) from tumors have a multicellular identity that more faithfully recapitulates the complexity of the tumors from which they were derived [7–9]. 2D cell cultures grow in a monolayer, which limits the interactions and environment experienced by cells in a real tumor. Tumor spheres have a 3D structure; however, they often lack the complex architecture, cellular heterogeneity, and variety of cell types found in PDOs. Tumor spheres are more homogeneous and may maintain some genetic features but often lose the heterogeneity and phenotypic characteristics of the original tumor over time. Therefore, tumor spheres may not fully recapitulate the original tumor's architecture compared to PDOs. From a cancer drug discovery perspective, tumor organoids’ multicellular identity more closely resembles tumors in vivo, significantly improving patient relevance and translatability, and representing a preclinical cancer model that may better replicate disease [10, 11]. While some evidence suggests that using patient-derived cancer organoids for pre-treatment drug testing may model the patient’s clinical response to chemotherapy [12–14], data regarding ovarian cancer patient-derived tumor organoids (OV-PDOs) are limited. However, recent reports suggest similar copy number alteration profiles compared with the parent tumors [11]. Using cancer organoid drug testing holds significant clinical relevance and has the potential to enhance the decision-making process in clinical settings.

## Methods and methods

### Patient enrollment

This study was approved by the institutional ethics committee of Taipei Medical University (Approval No. N201804045) and conducted in Shuang Ho Hospital, Taiwan (ROC). Informed consent was obtained from all participants and/or their legal guardians. All experiments of participants were performed in accordance with relevant guidelines and regulations. Clinical parameters including image studies, tumor markers, and outcomes were collected for analysis.

### Establishment and characterization of patient-derived organoids

For organoid preparation, tissues were obtained when appropriate surgeries were done with informed consent. Tumors were cleaned by PBS and dissociated by mixing the 0.5 U dispase (Merck, Darmstadt, Germany) with collagenase I (Thermo Fisher Scientific, Waltham, MA, USA) at 37 °C for 30–60 min. After digestion, it was centrifuged at 300 × g for 5 min at room temperature to deplete single cells. The cell pellet was suspended in Matrigel (Thermo Fisher Scientific), the Matrigel domes were solidified for 20 min before the culture medium was added. The culture medium was Advanced DMEM/F12 (Thermo Fisher Scientific) was supplemented with 2 mM HEPES (Cytiva, Marlborough, MA, USA), 1X GlutaMAX-I (Thermo Fisher Scientific), 1X B27 (Thermo Fisher Scientific), 1X N-2 supplement (Thermo Fisher Scientific), 10 ng/mL human EGF Recombinant Protein (Thermo Fisher Scientific), 100 ng/mL recombinant human FGF-10 (Thermo Fisher Scientific), 5% conditioned human Wnt3A medium (CRL-2647, ATCC, Manassas, VA, USA), 100 ng/mL human R-spondin (Sino Biological, Beijing, China), 100 ng/mL Noggin (Thermo Fisher Scientific), 200 U/mL penicillin/streptomycin (Thermo Fisher Scientific), and 9 µM Y-27632 (Merck), 1 mM nicotinamide (Merck), 500 nM SB431542 (Merck). Medium was exchanged every 2 to 3 days, and cultures were passaged at a 1:2–3 dilution every 1–4 weeks.

Organoid domes were mechanically disrupted by 0.5 U dispase for 1 h, washed by cold PBS, then fixed in 4% paraformaldehyde. Organoid pellet was embedded by histogel (Thermo Fisher Scientific). After paraffin embedding, tissues were sectioned and applied with standard H&E and IHC staining. IHC was performed using following primary antibodies with anti-TP53 (epredia, Kalamazoo, MI, USA), PAX8 (Cell Marque, Merck), WT1 (Roche, Basel, Switzerland), CK7 (epredia), CK20 (epredia), and Ki-67 (epredia) were diluted followed by data sheet, respectively. Tissue sections were incubated with a secondary antibody using the avidin–biotin peroxidase technique with DAKO Detection Kit (Agilent, Santa Clara, CA, USA). Images were acquired on a Leica microscope and processed using the Adobe Creative Cloud software package. The cell component of cancer organoids was reviewed by two pathologists.

### In vitro drug testing

Five thousand to ten thousand cells (~ 50 organoids) were seeded per well in a clear bottom, black 96 well plate (Thermo Fisher Scientific). Plates were incubated at 37 °C and 5% CO_2_ overnight. After seven days of drug treatment, the OV-PDOs viability were assessed by cell Titer-Glo^® ^3D Cell Viability Assay and normalized by the initial cell numbers. The guideline-recommended drugs, including carboplatin, epirubicin, gemcitabine, paclitaxel, and topotecan were evaluated. Each drug testing were performed in five replicates and presented as mean ± SEM.

### Data analysis

The data analyses were performed using GraphPad Prism software (Version 6.0, La Jolla, CA, USA). The dose response curve and area under the curve (AUC) were calculated based on cell viability percentage after treatment (untreated set as 1), the bigger the AUC value identified worse drug efficacy. All data with error bars are presented as mean ± SEM.

## Results

### Morphologic and molecular matching of patient-derived organoids to parent tumors

Thirty-eight patients with gynecological cancer were enrolled during 2020–2022. Tumors from 31 patients were successfully generated into organoids. Among them, 28 PDOs can be expanded, including 15 with ovarian cancer, 11 with endometrial cancer, and 2 with cervical cancer. Among the ovarian cancer PDOs, four were high-grade serous carcinoma (HGSC), four were mucinous, three were clear cells, one was carcinosarcoma, and one was endometrioid (Table 1).

**Table 1:** The characteristics of patients with ovarian cancer

Original ID	Sample ID	Cancer type	Age	FIGO stage	Neoadjuvant chemotherapy	Debulking status	Adjuvant chemotherapy	Survival status	BRCA geme status	Organoid culture	Organoid expansion
OvCa-19	HGSC-1	High grade serous	54	IIIC	Not received	Suboptimal	Paclitaxel and carboplatin x 6 cycles, followed by lipodox x12 cycles, then second debulking then paclitaxel and carboplatin x6 cycles	Alive	Wild type	Success	No
OvCa-21	EM-1	Endometrioid	40	IC1	Not received	Optimal	Paclitaxel and carboplatin x 9 cycles	Alive	Unexamined	Failed	-
OvCa-26	MC-1	Mucinous	37	IVB	Not received	Optimal	Paclitaxel and carboplatin x 6 cycles, followed by lipodox x 3 cycles, then gemcitabine and carboplatin x3 cycles	Expired	Unexamined	Success	Yes
OvCa-28	MC-2	Mucinous	66	IIIA	Not received	Optimal	Not received due to acute stroke	Expired	Unexamined	Success	Yes
OvCa-29	HGSC-2	High grade serous	61	IVB	Not received	Suboptimal	Paclitaxel and carboplatin x 4 cycles, followed by interval debulking, then paclitaxel and carboplatin x 6 cycles, then lipodox, carboplatin and bevacizumab x6 cycles	Alive	Wild type	Success	Yes
OvCa-32	HGSC-3	High grade serous	62	IVB	Not received	Suboptimal	Paclitaxel and carboplatin x 4 cycles, followed by interval debulking, then paclitaxel and carboplatin x 6 cycles, then lipodox, carboplatin and bevacizumab x6 cycles	Alive	Wild type	Failed	-
OvCa-33	HGSC-4	High grade serous	41	IIIC	Not received	Suboptimal	Paclitaxel and carboplatin x 6 cycles, then lipodox x 6 cycles	Alive	Mutation	Success	Yes
OvCa-34	MC-3	Mucinous	43	IC1	Not received	Optimal	Paclitaxel and cisplatin x4 cycles, then lipodox and bevacizumab x 5 cycles, then gemcitabine and bevacizumab x 2 cycles, then topotecan x 8 cycles	Expired	Unexamined	Failed	-
OvCa-37	CCSC-1	Carcinosarcoma	61	IIIC	Not received	Optimal	Paclitaxel, carboplatin and bevacizumab x 9 cycles, followed by second debulking, then bevacizumab, paclitaxel and carboplatin x 6 cycles, then lipodox x 3 cycles, then gemcitabine x2 cycles	Expired	Wild type	Success	Yes
OvCa-38	CCC-1	Clear cell	32	IC1	Not received	Optimal	Paclitaxel and carboplatin x 4 cycles	Alive	Unexamined	Success	Yes
OvCa-40	CCC-2	Clear cell	49	IIIC	Bevacizumab, paclitaxel and cisplatin x 3 cycle then paclitaxel and carboplatin x 2 cycle	Suboptimal	Gemcitabine and carboplatin x1 cycle	Expired	Unexamined	Success	Yes
OvCa-41	CCC-3	Clear cell	50	IIIB	Not received	Optimal	Rejected	Expired	Unexamined	Success	Yes
OvCa-42	MC-4	Mucinous	47	IC1	Not received	Optimal	Not received	Alive	Unexamined	Success	Yes
OvCa-43	HGSC-5	High grade serous	55	IIIC	Paclitaxel and carboplatin x 4 cycle	Optimal	Paclitaxel and carboplatin x 2 cycles, followed by lipodox x 3 cycles, then gemcitabine x3 cycle	Expired	Wild type	Success	Yes
OvCa-44	EM-2	Endometrioid	58	IIB	Not received	Suboptimal	Paclitaxel and cisplatin x 6 cycles, then lipodox x 3 cycles, then gemcitabine and cisplatin x2 cycles, followed by secondary optimal debulking, then gemcitabine and cisplatin x2 cycles	Alive	Not received	Success	Yes
OvCa-45	HGSC-6	High grade serous	70	IIIC	Not received	Optimal	Paclitaxel and carboplatin x 6 cycles, then lipodox and carboplatin x4 cycles	Alive	Unexamined	Success	No
OvCa-46	MC-5	Mucinous	44	IC1	Not received	Optimal	Not received	Alive	Unexamined	Success	Yes
OvCa-49	HGSC-7	High grade serous	55	IIIC	Paclitaxel and cisplatin x 4 cycle	Optimal	Paclitaxel and carboplatin x 2 cycles, followed by lipodox x 3 cycles, then gemcitabine x3 cycles	Expired	Wild type	Success	Yes
OvCa-50	CCC-4	Clear cell	45	IIIC	Not received	Optimal	Paclitaxel and carboplatin x 1 cycle, then second debulking surgery, followed by lipodox and bevacizumab x 1 cycle, then gemcitabine, carboplatin and bevacizumab x 8 cycles.	Alive	Unexamined	Success	Yes
OvCa-51	CCSC-2	Carcinosarcoma	51	IIIA2	Not received	Optimal	Paclitaxel and cisplatin x 1 cycle, then gemcitabine and paclitaxel x 3 cycles	Expired	Unexamined	Failed	-
OvCa-54	CCC-6	Clear cell	51	IIB	Not received	Optimal	Paclitaxel and cisplatin x 6 cycles	Alive	Unexamined	Success	Yes

**Fig. 1:**
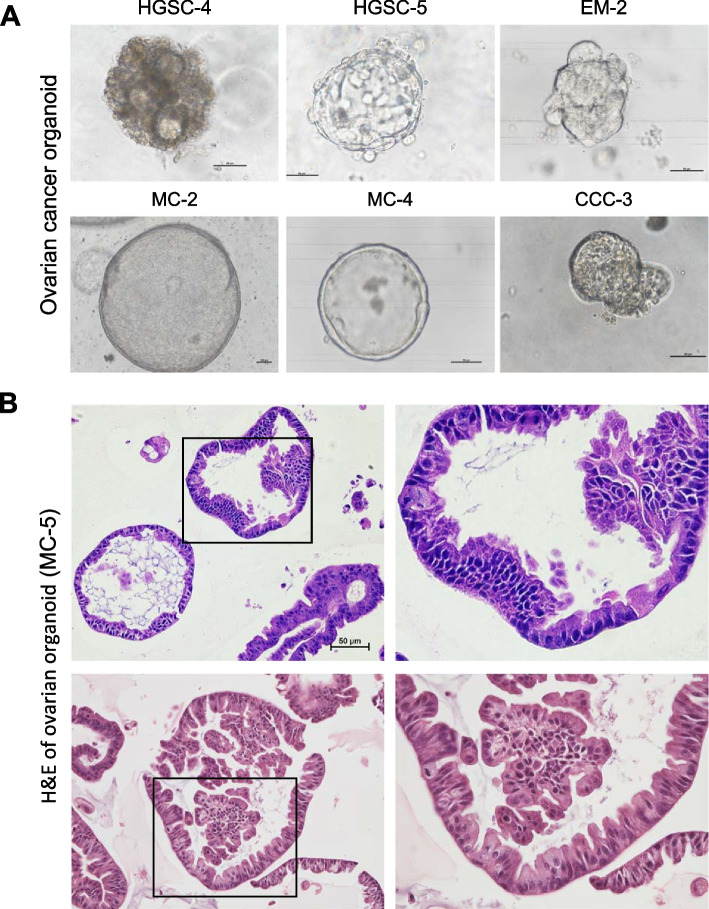
OV-PDOs show interpatient morphology differences. **A** Phase-contrast of PDOs under culture with matrix gel and appropriate growth factors. **B** H&E staining of PDOs shows epithelial invaginations and folding as well as a round, cystic phenotype with lumen formation

Different pathologic types presented with different appearances (Fig. 1A). HGSC organoids displayed papillary branching, with a glandular-like protruding contour. Endometrioid organoids showed a glandular pattern with smaller cells and more confluent surface. Mucinous cancer organoids showed vanished glandular architecture and a simple, non-stratified cell lining outside with mucin-like content. Clear cell cancer organoids had polyhedral, flattened cells with vacuolated cytoplasm.

As shown in Fig. 1A, two HGSC organoids (HGSC-4 and HGSC-5) from different patients displayed different cell densities and arrangements, suggesting its interpatient heterogeneity. Interestingly, within a single patient, organoid sizes, cellular densities, and protruding contours were distinct; these morphologic differences thus also indicate intrapatient heterogeneity (HGSC-4, HGSC-5, and HGSC-7, Supplementary Figure S1).

With hematoxylin and eosin (H&E) staining, mucinous organoid cells were spheroid with the cell apical at inner side and basal at outer side. The organoid cells had a high nuclear-cytoplasmic ratio, and nuclear polymorphism (Fig. 1B). High-power field showed that organoid cells had vacuolated and secretions. We further assessed organoid biomolecular preservation with H&E and immunohistochemical staining of organoids, parent tumors, and Human Protein Atlas (https://www.proteinatlas.org/) references.

**Fig. 2:**
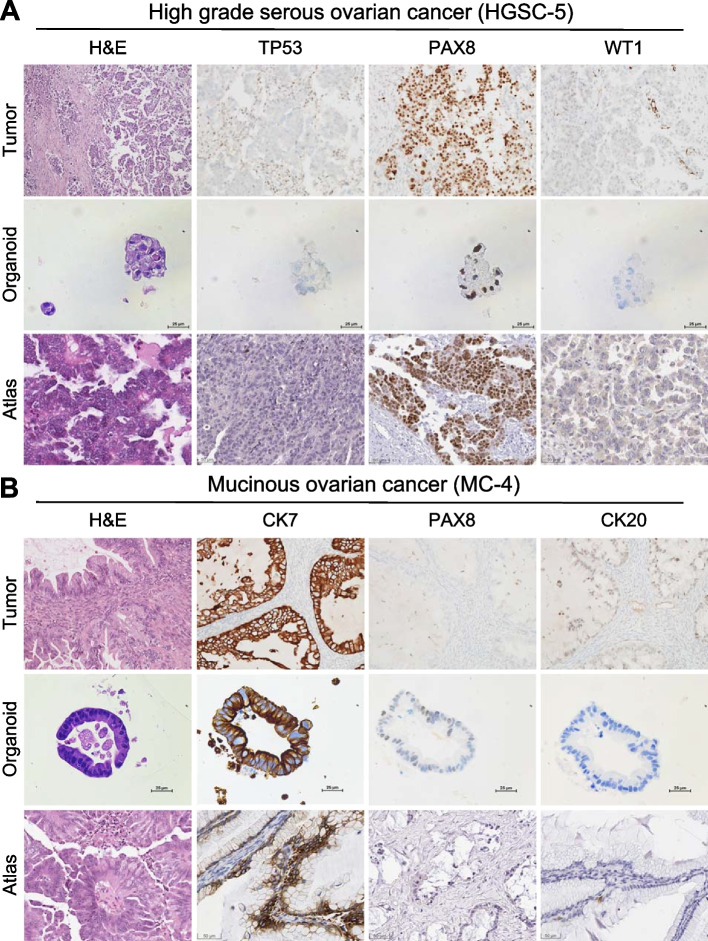
Patient-derived organoids morphologically and molecularly matched the parent tumors. H&E stain and immunohistochemistry of PDOs in HGSC (HGSC-5, **A**) and mucinous ovarian cancer (MC-4, **B**) in paired tumor (upper), OV-PDOs (middle), and database (lower)

The characteristics of organoids and parental tumors were compared. We presented case HGSC-5, which is a p53 null-expression patient. As shown in Fig. 2A, the organoids displayed a high nuclear-cytoplasmic ratio, irregular nuclei, and prominent nucleoli, recapitulating all malignancy features. p53 immunostain showed null expression in HGSC PDOs and was matched to the parent tumors. In addition, the strong positive PAX8 and weak positive WT1 were compatible with parent tumors and a p53 null-expression case in the Human Protein Atlas. In primary mucinous ovarian cancer, the immunohistochemistry expression of PAX8 is usually low [15]. Mucinous carcinoma PDOs presented with diffuse CK7 positive, focal PAX8 positive, and CK20 negative, matching the parent tumors and reference data (Fig. 2B). This cumulative evidence indicates the successful establishment of ovarian cancer-derived organoids, of which the morphology and molecular characteristics were consistent with parent tissues.

### Heterogenous chemosensitivity profile of ovarian *cancer* organoids

**Fig. 3:**
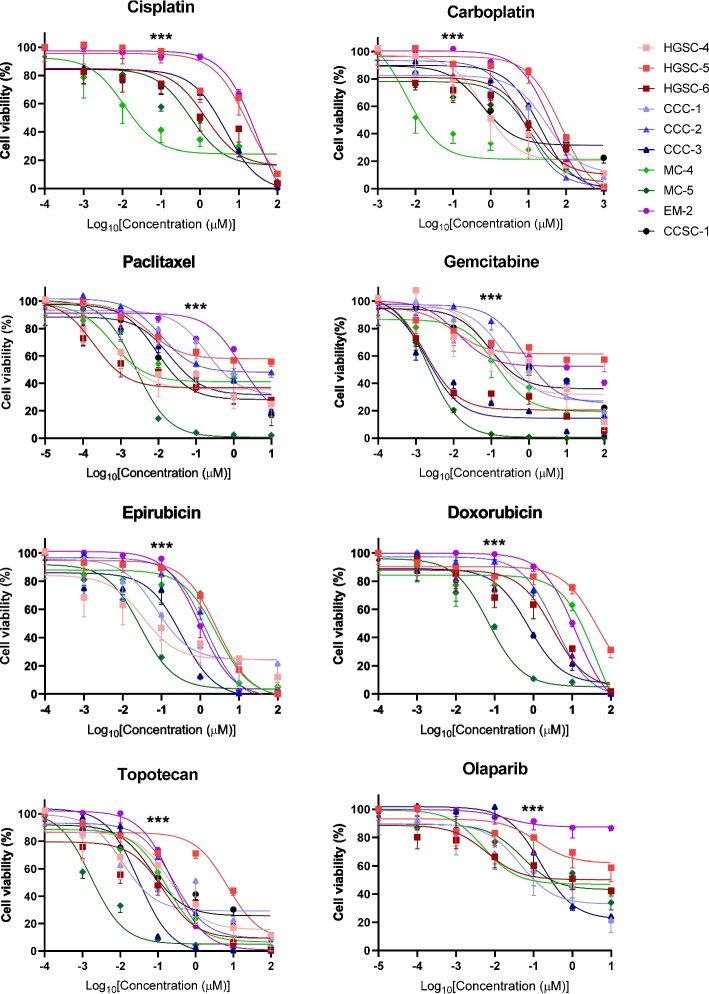
Drug testing and personalized therapy of ovarian cancer in OV-PDOs. Dose–response curves of 10 OV-PDOs treated with cisplatin, carboplatin, paclitaxel, gemcitabine, epirubicin, doxorubicin, topotecan, and olaparib. Dots represent five-repetition means. Error bars represent five-repetition standard error of the mean. The statistical analysis of drug response at 0.1 µM was calculated using the chi-square test

**Fig. 4:**
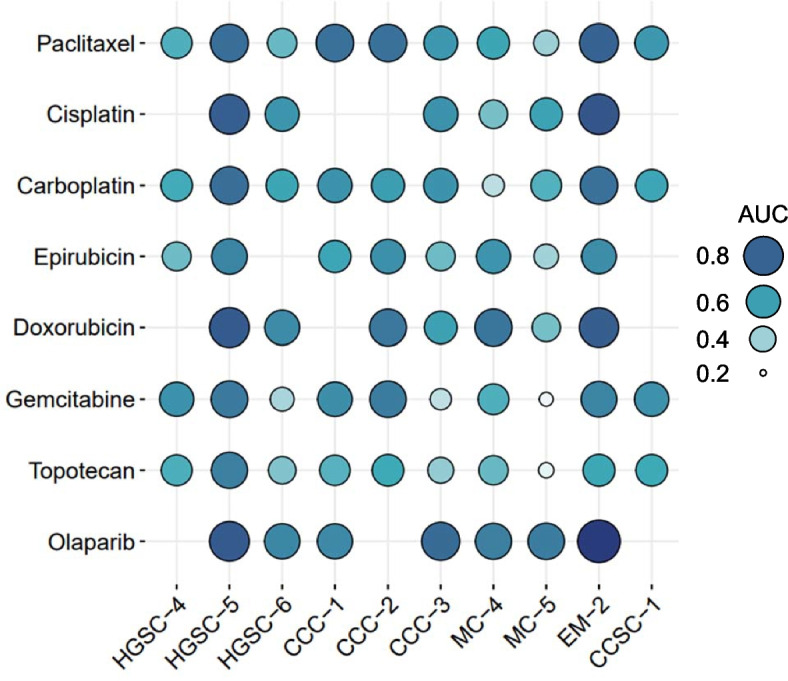
Area under the drug response curve values mapped to the balloon plot. AUC for a fixed concentration range. Circle color and size indicates AUC results. AUC can be seen as average efficacy and compared across patients

We tested drug responses in 10 OV-PDOs using guideline-recommended chemotherapeutic agents, including paclitaxel, cisplatin, carboplatin, epirubicin, doxorubicin, gemcitabine, topotecan, and olaparib. The drug sensitivity analysis is presented in Fig. 3 and estimated IC_50_ values are shown in Supplementary Figure S2. The areas under the dose–response curves for each drug were also calculated (Fig. 4). Each PDO had a unique dose–response curve for each drug, indicating interpatient heterogeneity even within the same histology type. A lower area under the curve (AUC) indicates a more sensitive response, as demonstrated in Fig. 4 (smaller circles indicate better drug choices).

The HGSC-4 OV-PDO was relatively sensitive to epirubicin (AUC = 0.496) and topotecan (AUC = 0.550). The HGSC-6 OV-PDO was relatively sensitive to paclitaxel (AUC = 0.507), gemcitabine (AUC = 0.382), and topotecan (AUC = 0.461). The HGSC-5 OV-PDO responded poorly to all drugs (AUC = 0.691–0.831). For the clear cell ovarian cancer (CCC) OV-PDOs, three were more sensitive to topotecan (AUC = 0.425–0.570) and CCC-3 was more sensitive to gemcitabine (AUC = 0.335). Between the two mucinous OV-PDOs, MC-5 was more sensitive to gemcitabine (AUC = 0.239) and topotecan (AUC = 0.253). These results demonstrate an OV-PDOs-guided precision therapy approach.

### Clinical relevance of organoid drug testing

**Fig. 5:**
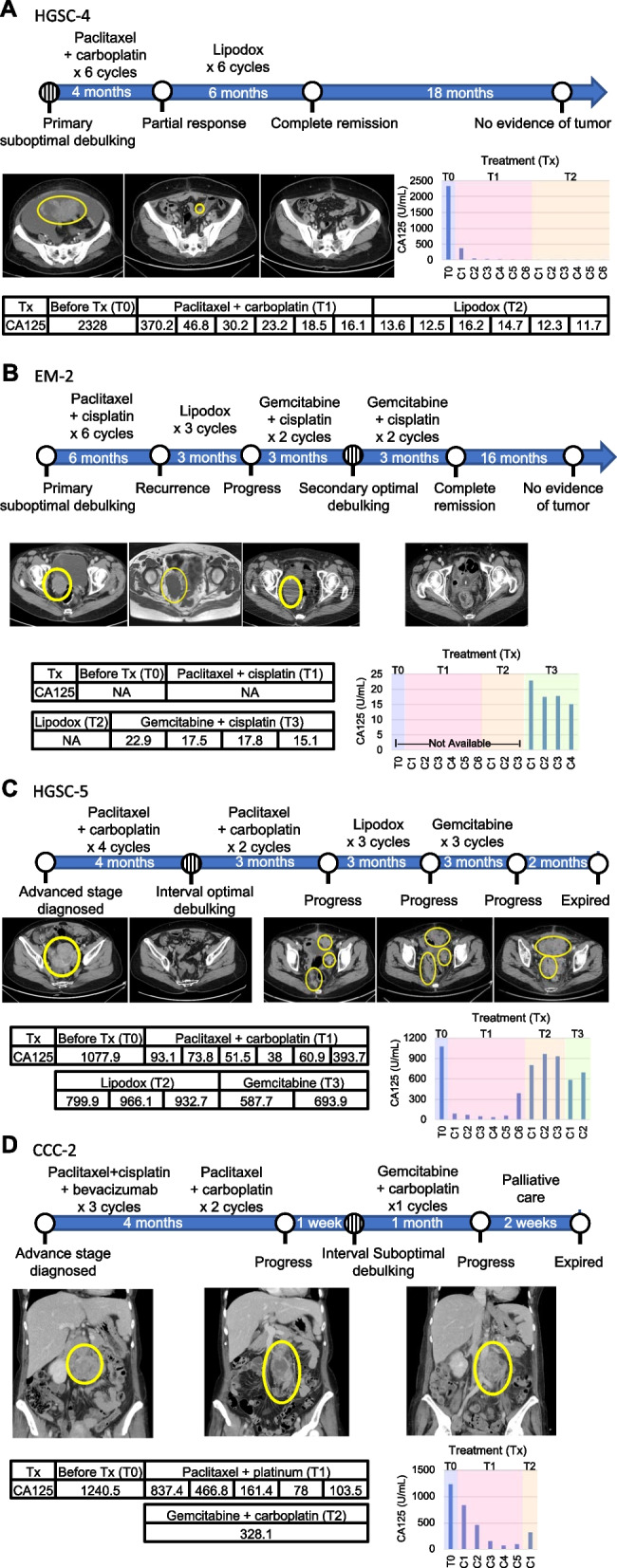
Drug sensitivity compatibility between PDOs and clinical data. Summary timeline of the platinum-sensitive (HGSC-4, **A**), resistant (EM-2, **B**), and refractory (HGSC-5, **C**; CCC-2 **D**) treatment plans. The reference range of CA 125 is 0–35 units/mL. Circle with straight lines indicates the time of sample collection

The clinical courses of four patients, who included platinum-sensitive, resistant, and refractory profiles, were correlated with their OV-PDOs drug response results (Fig. 5).

Patient HGSC-4 had stage IIIC platinum-sensitive HGSC and had undergone primary suboptimal debulking surgery (Fig. 5A). After surgery, she received adjuvant chemotherapy with paclitaxel and carboplatin, based on guideline recommendations. The tumor markers decreased and the clinical image revealed partial tumor response after six cycles. Because of intolerable neurological toxicity, especially hand numbness, the regiment was changed to liposomal doxorubicin (Lipodox). After six cycles of Lipodox, she was tumor-free. There was no tumor recurrence during the following 18 months. In vitro drug testing revealed that this patient’s OV-PDOs (Fig. 4, HGSC-4) were sensitive to taxanes (paclitaxel, AUC = 0.546), platinum (carboplatin, AUC = 0.565), and anthracycline (epirubicin, AUC = 0.496), which is compatible with her clinical course.

Patient EM-2 had platinum-resistant, stage IIB, dedifferentiated endometrioid ovarian cancer. She had a suboptimal debulking surgery following adjuvant chemotherapy, with six cycles of paclitaxel and carboplatin to achieve complete remission (Fig. 5B). After four months, tumor recurrence at the presacral area was found. She had chemotherapy with three cycles of Lipodox, but the tumor progressed. She then underwent chemotherapy with gemcitabine and cisplatin, and a second, optimal debulking operation. There was no evidence of disease for 16 months (at the time of manuscript preparation). Her OV-PDOs (Fig. 4, EM-2), derived at the second debulking surgery, showed resistance to paclitaxel (AUC = 0.802), cisplatin (AUC = 0.846) and Lipodox (doxorubicin, AUC = 0.820) and relative sensitivity to gemcitabine (AUC = 0.692) and topotecan (AUC = 0.578). Thus, topotecan may be a better drug choice in the event of future recurrence.

Patient HGSC-5 had platinum-refractory, stage IIIC HGSC. She received four cycles of neoadjuvant chemotherapy with paclitaxel and carboplatin, followed by optimal interval debulking (Fig. 5C). Tumor progression developed after two further cycles of paclitaxel and carboplatin. Chemotherapy was then shifted to Lipodox. Tumor progression occurred again after three cycles of Lipodox, at which time gemcitabine was administered. The tumor still progressed after three cycles. The patient began palliative care and expired a few months later. Her OV-PDO drug tests (Fig. 4, HGSC-5) revealed multiple drug resistances to these chemotherapeutic agents (paclitaxel, AUC = 0.765; carboplatin, AUC = 0.766; doxorubicin, AUC = 0.831; gemcitabine, AUC = 0.728).

Patient CCC-2 had platinum-refractory, stage IIIC CCC. Metastatic para-aortic lymph node was found by the general surgeon, and she received three cycles of neoadjuvant treatment with bevacizumab, paclitaxel, and cisplatin at the gynecologic department (Fig. 5D). Because of proteinuria and nephrotoxicity, she had paclitaxel and carboplatin for two more cycles. Imaging showed a growing para-aortic tumor; therefore, she underwent interval suboptimal debulking surgery. She received adjuvant chemotherapy with gemcitabine and carboplatin. However, she was found to have jaundice before the next chemotherapy cycles, and imaging revealed rapid tumor growth. Family counseling led to the decision to take palliative care for the rest of her life, and she expired two weeks later. Her OV-PDOs (Fig. 4, CCC-2), derived at the interval debulking surgery, presented relative resistance to paclitaxel (AUC = 0.754), carboplatin (AUC = 0.610), and gemcitabine (AUC = 0.718).

## Discussion

The feasibility of in vitro drug testing using OV-PDOs in a clinical setting could be based on several key parameters: (1) Establishment success rate—the ability to successfully establish PDOs from patient samples; (2) Representativeness—the extent to which PDOs retain the histological characteristics of the original tumor; (3) Drug response correlation—the ability of PDOs to mimic patient-specific drug responses observed in clinical settings Our results demonstrated that PDOs can be established, expanded, and exhibited characteristics similar to the original tumors. Additionally, the organoid drug testing results were consistent with the clinical responses of patients who were platinum sensitive, resistant, and refractory. Further development of cancer tissue-derived organoids as a platform for drug selection may improve future precision of chemotherapy treatments for gynecological cancers.

In the patient who was platinum-sensitive, her OV-PDO (HGSC-4) was compatible with a chemo-sensitive tumor profile; she was tumor-free for a year and a half after chemotherapy. In the future, preclinical testing may give patients greater confidence when they begin chemotherapy. In the patient who was platinum-resistant, her OV-PDO (EM-2) results showed multidrug resistance, but relative sensitivity to gemcitabine. Her clinical data confirmed the efficiency of gemcitabine and that the organoids from treated tumors present drug responses consistent with those of the parent tumor. Knowing drug test results preclinically may lead to choosing first-line chemotherapy other than taxane and platinum agents in routine care. In patients who were platinum-refractory (HGSC-5 and CCC-2), multidrug resistance predictions may facilitate better clinical treatment decisions, including use of rare chemotherapeutic agents and/or avoidance of end-stage side-effects. Our results thus contribute to the potential for PDOs in ovarian precision medicine, especially in the recurrent or refractory setting, providing both patients and doctors more information prior to chemotherapy.

There are burgeoning efforts toward using OV-PDOs for drug selection. OV-PDO collections provide an opportunity to retain intratumor heterogeneity and to repeatedly test the comprehensive genotype–phenotype correlations. Indeed, the genetic background, including copy number variation and single nucleotide variants in ovarian cancer organoids are similar to cancer tissues [4, 10, 11]. The carboplatin resistance pattern has also been found to be consistent between OV-PDOs and human tumors [16]. In generated BRCA1 and BRCA2 mutation mouse ovarian organoids, drug testing predicted olaparib-sensitivity [16]. This cumulative evidence supports the conclusion that OV-PDOs may mimic drug responses in vivo.

As a supplement to guideline-recommended regimens, OV-PDOs may provide a platform for repurposing or investigating new drugs. A few studies have applied DNA repair inhibitors (PARP inhibitor rucaparib) to OV-PDOs, showing the capability of organoids as a drug test model for targeted therapies [16, 17]. Short-term OV-PDOs have also been used to explore prediction responses to DNA repair inhibitors; these investigators tested 22 OV-PDOs with a panel of DNA repair inhibitors and found that organoids with mutations in homologous recombination DNA repair genes were more sensitive to DNA repair inhibitors (CHK1 inhibitor prexasertib and ATR inhibitor VE-822) than those without mutations [17]. They also identified potential biomarkers of drug response and resistance, which may help guide personalized treatments. In another study, OVPDOs were used to identify miRNA interactions with ovarian cancer cells; wide-ranging tumor suppressor effects of specific miRNA were found, and the combination of epidermal growth factor receptor (EGFR) inhibitor had cytotoxic effects on OV-PDOs [18]. These studies suggest that PDO drug testing can provide valuable information for identifying novel drugs and drug combinations, discovering potential biomarkers of response, guiding treatment decisions, and improving clinical outcomes of cancer treatment.

In other cancer types, development of uses for PDOs in personalized treatment are ongoing. In pancreatic cancer [14], PDO drug sensitivity is highly correlated with clinical response to treatment. In metastatic gastrointestinal cancers, organoid drug sensitivity was predictive of clinical response in a subset of patients [13]. In breast cancer [19], PDO drug sensitivity is significantly associated with patient response to treatment in targeted therapy and chemotherapy. In lung cancer, PDOs have shown clinical correlations when tested in response to olaparib, anti-EGFR targeted therapy, and different chemotherapeutic agents [20]. In locally advanced colorectal cancer, a PDO model showed chemoradiation sensitivity and was correlated with patient response with an accuracy rate reaching 84.43% [12]. By contrast, there are limited results from PDO application to ovarian cancers. Our results herein support PDO use as a drug selection platform for patients with ovarian cancer.

While our study highlights the potential of PDOs for personalized chemotherapy, there are several limitations in the present study should be addressed in the future. First, the long-term stability and genetic fidelity of PDOs need to be assessed in larger-scale studies to confirm reproducibility and reliability across diverse patient populations. Integration with other molecular and genomic profiling techniques could enhance predictive accuracy. Second, the histotype classification was based on H&E staining and limited markers by IHC. More comprehensive IHC analyses may further clarify the heterogeneity among different histotypes. Third, further evaluation of the clinical utility of PDOs for drug selection in endometrial and cervical tumors would extend the applicability of this platform to a broader range of gynecological cancers.

## Conclusion

Herein, we established ovarian cancer PDOs as an in vitro drug testing platform. A PDO-based drug test-guided trial is now warranted, to refine current treatment recommendations. Further investigations of PDOs-based chemotherapy, target therapy, immunotherapy, and cell therapy may shed new light on future precision ovarian cancer treatments.

## Supplementary information

Supplementary Material 1.

## Data Availability

The datasets generated during and/or analyzed during the current study are available from the corresponding author upon reasonable request. All the generated data are given here as Figures, Table, and supplementary material. Personal information will not be provided to ensure the anonymity of the patient.

## Abbreviations

CTCs: Circulating tumor cells
PDOs: Patient-derived organoids
PDX: Patient-derived xenograft
CNA: Copy number alteration
AUC: Area under the curve
CCC: Clear cell ovarian cancer
HGSC: High-grade serous carcinoma

## Data availability

No datasets were generated or analysed during the current study.
