# Correction: In vitro drug testing using patient-derived ovarian cancer organoids

**DOI:** 10.1186/s13048-024-01559-1

**Published:** 2024-12-05

**Authors:** Lin-Yu Chen, Yu-Ting Chou, Phui-Ly Liew, Ling-Hui Chu, Kuo-Chang Wen, Shiou-Fu Lin, Yu-Chun Weng, Hui-Chen Wang, Po-Hsuan Su, Hung-Cheng Lai

**Affiliations:** 1 Department of Obstetrics and Gynecology, Shuang Ho Hospital, Taipei Medical University, New Taipei City, 23561 Taiwan; 2 Department of Pathology, Shuang Ho Hospital, Taipei Medical University, New Taipei City, 23561 Taiwan; 3 Department of Pathology, School of Medicine, College of Medicine, Taipei Medical University, Taipei, 11031 Taiwan; 4 Department of Obstetrics and Gynecology, School of Medicine, College of Medicine, Taipei Medical University, Taipei, 11031 Taiwan; 5 Translational Epigenetics Center, Shuang Ho Hospital, Taipei Medical University, New Taipei City, 23561 Taiwan; 6 Department of Obstetrics and Gynecology, Tri-Service General Hospital, National Defense Medical Center, Taipei, 11490 Taiwan; 7 College of Health Technology, National Taipei University of Nursing and Health Sciences, Taipei, 11219 Taiwan

**Correction: J Ovarian Res 17**, **194 (2024)**

10.1186/s13048-024-01520-2

Following publication of the original article [1], the authors identified an error in Table 1 caption and content.

**Incorrect table**.

**Table 1:** The characteristics of patients with ovarian cancer (V5)

Adjuvant chemotherapy	Survival status	BRCA geme status	Organoid culture	Organoid expansion
Paclitaxel and carboplatin × 6 cycles, followed by lipodox × 12 cycles, then second debulking then paclitaxel and carboplatin × 6 cycles	Alive	Wild type	Success	No
Paclitaxel and carboplatin × 9 cycles	Alive	Unexamined	Failed	-
Paclitaxel and carboplatin × 6 cycles, followed by lipodox × 3 cycles, then gemcitabine and carboplatin × 3 cycles	Expired	Unexamined	Success	Yes
Not received due to acute stroke	Expired	Unexamined	Success	Yes
Paclitaxel and carboplatin × 4 cycles, followed by interval debulking, then paclitaxel and carboplatin × 6 cycles, then lipodox, carboplatin and bevacizumab × 6 cycles	Alive	Wild type	Success	Yes
Paclitaxel and carboplatin × 4 cycles, followed by interval debulking, then paclitaxel and carboplatin × 6 cycles, then lipodox, carboplatin and bevacizumab × 6 cycles	Alive	Wild type	Failed	-
Paclitaxel and carboplatin × 6 cycles, then lipodox × 6 cycles	Alive	Mutation	Success	Yes
Paclitaxel and cisplatin × 4 cycles, then lipodox and bevacizumab × 5 cycles, then gemcitabine and bevacizumab × 2 cycles, then topotecan × 8 cycles	Expired	Unexamined	Failed	-
Paclitaxel, carboplatin and bevacizumab × 9 cycles, followed by second debulking, then bevacizumab, paclitaxel and carboplatin × 6 cycles, then lipodox × 3 cycles, then gemcitabine × 2 cycles	Expired	Wild type	Success	Yes
Paclitaxel and carboplatin × 4 cycles	Alive	Unexamined	Success	Yes
Gemcitabine and carboplatin × 1 cycle	Expired	Unexamined	Success	Yes
Rejected	Expired	Unexamined	Success	Yes
Not received	Alive	Unexamined	Success	Yes
Paclitaxel and carboplatin × 2 cycles, followed by lipodox × 3 cycles, then gemcitabine × 3 cycle	Expired	Wild type	Success	Yes
Paclitaxel and cisplatin × 6 cycles, then lipodox × 3 cycles, then gemcitabine and cisplatin × 2 cycles, followed by secondary optimal debulking, then gemcitabine and cisplatin × 2 cycles	Alive	Not received	Success	Yes
Paclitaxel and carboplatin × 6 cycles, then lipodox and carboplatin × 4 cycles	Alive	Unexamined	Success	No
Not received	Alive	Unexamined	Success	Yes
Paclitaxel and carboplatin × 2 cycles, followed by lipodox × 3 cycles, then gemcitabine × 3 cycles	Expired	Wild type	Success	Yes
Paclitaxel and carboplatin × 1 cycle, then second debulking surgery, followed by lipodox and bevacizumab × 1 cycle, then gemcitabine, carboplatin and bevacizumab × 8 cycles	Alive	Unexamined	Success	Yes
Paclitaxel and cisplatin × 1 cycle, then gemcitabine and paclitaxel × 3 cycles	Expired	Unexamined	Failed	-
Paclitaxel and cisplatin × 6 cycles	Alive	Unexamined	Success	Yes

**Correct table**.

**Table 1:** The characteristics of patients with ovarian cancer

Original ID	Sample ID	Cancer type	Age	FIGO stage	Neoadjuvant chemotherapy	Debulking status	Adjuvant chemotherapy	Survival status	BRCA geme status	Organoid culture	Organoid expansion
OvCa-19	HGSC-1	High grade serous	54	IIIC	Not received	Suboptimal	Paclitaxel and carboplatin x 6 cycles, followed by lipodox x12 cycles, then second debulking then paclitaxel and carboplatin x6 cycles	Alive	Wild type	Success	No
OvCa-21	EM-1	Endometrioid	40	IC1	Not received	Optimal	Paclitaxel and carboplatin x 9 cycles	Alive	Unexamined	Failed	-
OvCa-26	MC-1	Mucinous	37	IVB	Not received	Optimal	Paclitaxel and carboplatin x 6 cycles, followed by lipodox x 3 cycles, then gemcitabine and carboplatin x3 cycles	Expired	Unexamined	Success	Yes
OvCa-28	MC-2	Mucinous	66	IIIA	Not received	Optimal	Not received due to acute stroke	Expired	Unexamined	Success	Yes
OvCa-29	HGSC-2	High grade serous	61	IVB	Not received	Suboptimal	Paclitaxel and carboplatin x 4 cycles, followed by interval debulking, then paclitaxel and carboplatin x 6 cycles, then lipodox, carboplatin and bevacizumab x6 cycles	Alive	Wild type	Success	Yes
OvCa-32	HGSC-3	High grade serous	62	IVB	Not received	Suboptimal	Paclitaxel and carboplatin x 4 cycles, followed by interval debulking, then paclitaxel and carboplatin x 6 cycles, then lipodox, carboplatin and bevacizumab x6 cycles	Alive	Wild type	Failed	-
OvCa-33	HGSC-4	High grade serous	41	IIIC	Not received	Suboptimal	Paclitaxel and carboplatin x 6 cycles, then lipodox x 6 cycles	Alive	Mutation	Success	Yes
OvCa-34	MC-3	Mucinous	43	IC1	Not received	Optimal	Paclitaxel and cisplatin x4 cycles, then lipodox and bevacizumab x 5 cycles, then gemcitabine and bevacizumab x 2 cycles, then topotecan x 8 cycles	Expired	Unexamined	Failed	-
OvCa-37	CCSC-1	Carcinosarcoma	61	IIIC	Not received	Optimal	Paclitaxel, carboplatin and bevacizumab x 9 cycles, followed by second debulking, then bevacizumab, paclitaxel and carboplatin x 6 cycles, then lipodox x 3 cycles, then gemcitabine x2 cycles	Expired	Wild type	Success	Yes
OvCa-38	CCC-1	Clear cell	32	IC1	Not received	Optimal	Paclitaxel and carboplatin x 4 cycles	Alive	Unexamined	Success	Yes
OvCa-40	CCC-2	Clear cell	49	IIIC	Bevacizumab, paclitaxel and cisplatin x 3 cycle then paclitaxel and carboplatin x 2 cycle	Suboptimal	Gemcitabine and carboplatin x1 cycle	Expired	Unexamined	Success	Yes
OvCa-41	CCC-3	Clear cell	50	IIIB	Not received	Optimal	Rejected	Expired	Unexamined	Success	Yes
OvCa-42	MC-4	Mucinous	47	IC1	Not received	Optimal	Not received	Alive	Unexamined	Success	Yes
OvCa-43	HGSC-5	High grade serous	55	IIIC	Paclitaxel and carboplatin x 4 cycle	Optimal	Paclitaxel and carboplatin x 2 cycles, followed by lipodox x 3 cycles, then gemcitabine x3 cycle	Expired	Wild type	Success	Yes
OvCa-44	EM-2	Endometrioid	58	IIB	Not received	Suboptimal	Paclitaxel and cisplatin x 6 cycles, then lipodox x 3 cycles, then gemcitabine and cisplatin x2 cycles, followed by secondary optimal debulking, then gemcitabine and cisplatin x2 cycles	Alive	Unexamined	Success	Yes
OvCa-45	HGSC-6	High grade serous	70	IIIC	Not received	Optimal	Paclitaxel and carboplatin x 6 cycles, then lipodox and carboplatin x4 cycles	Alive	Unexamined	Success	No
OvCa-46	MC-5	Mucinous	44	IC1	Not received	Optimal	Not received	Alive	Unexamined	Success	Yes
OvCa-49	HGSC-7	High grade serous	55	IIIC	Paclitaxel and cisplatin x 4 cycle	Optimal	Paclitaxel and carboplatin x 2 cycles, followed by lipodox x 3 cycles, then gemcitabine x3 cycles	Expired	Wild type	Success	Yes
OvCa-50	CCC-4	Clear cell	45	IIIC	Not received	Optimal	Paclitaxel and carboplatin x 1 cycle, then second debulking surgery, followed by lipodox and bevacizumab x 1 cycle, then gemcitabine, carboplatin and bevacizumab x 8 cycles.	Alive	Unexamined	Success	Yes
OvCa-51	CCSC-2	Carcinosarcoma	51	IIIA2	Not received	Optimal	Paclitaxel and cisplatin x 1 cycle, then gemcitabine and paclitaxel x 3 cycles	Expired	Unexamined	Failed	-
OvCa-54	CCC-6	Clear cell	51	IIB	Not received	Optimal	Paclitaxel and cisplatin x 6 cycles	Alive	Unexamined	Success	Yes

The original article [1] has been corrected.
