# Supplementary material for: In vitro drug testing using patient-derived ovarian cancer organoids
Source: J Ovarian Res. 2024 Oct 2;17:194. doi: 10.1186/s13048-024-01520-2 (PMC11445862; doi:10.1186/s13048-024-01520-2)
Supplement: Supplementary file 1 — Supplementary Material 1. [file 13048_2024_1520_MOESM1_ESM.pdf]

Figure S1

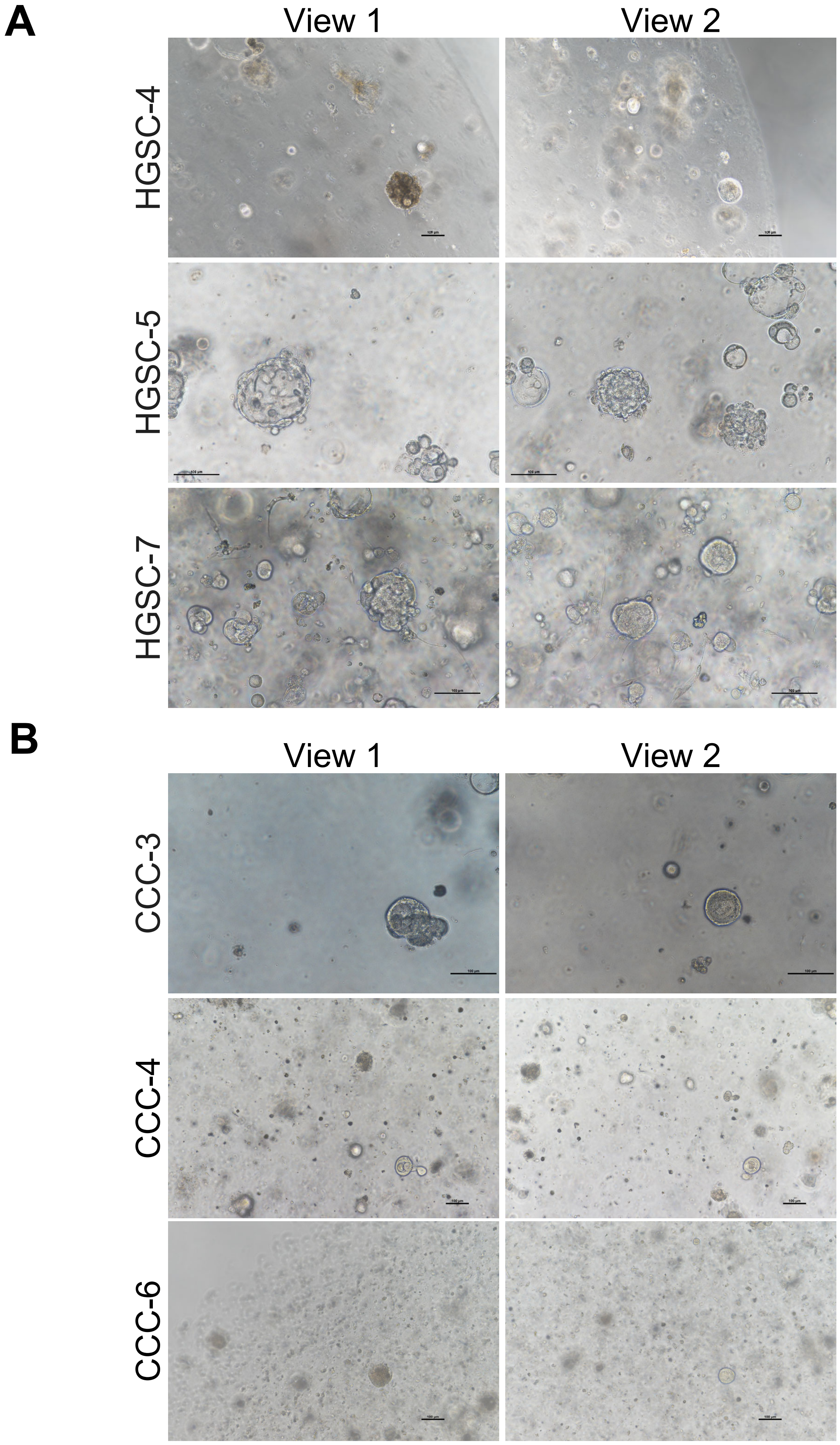

Figure S2

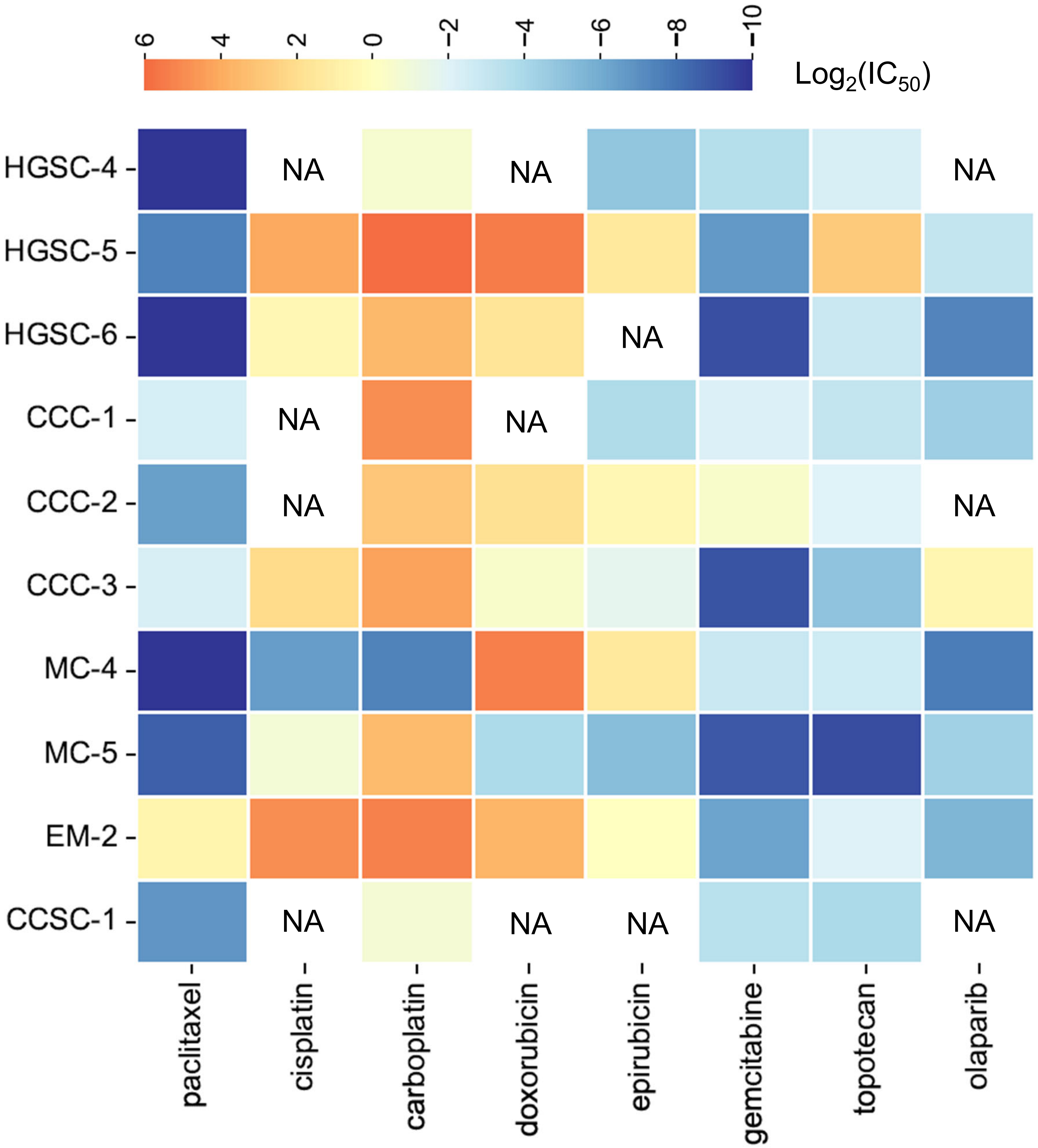
6
4
2
0
−2
−4
−6
−8
−10
$Log_2(IC_{50})$
HGSC-4
HGSC-5
HGSC-6
CCC-1
CCC-2
CCC-3
MC-4
MC-5
EM-2
CCSC-1
NA
paclitaxel
cisplatin
carboplatin
doxorubicin
epirubicin
gemcitabine
topotecan
olaparib

Supplementary Figure legends

Figure S1. OV-PDOs exhibit intrapatient heterogeneity. Phase-contrast imaging of three HGSC PDOs (A) and three CCC PDOs (B) revealed distinct morphologies among organoids from the same patient, indicating intrapatient heterogeneity.

Figure S2. Visualization of drug IC_{50} values in ovarian cancer. Heat map display of estimated IC_{50} values, which represent the drug concentration required to inhibit 50% of the target response. Color intensity indicates IC_{50} values for different drugs.
